# Vaccination with Recombinant Subolesin Antigens Provides Cross-Tick Species Protection in *Bos indicus* and Crossbred Cattle in Uganda

**DOI:** 10.3390/vaccines8020319

**Published:** 2020-06-18

**Authors:** Paul D. Kasaija, Marinela Contreras, Fredrick Kabi, Swidiq Mugerwa, José de la Fuente

**Affiliations:** 1 SaBio, Instituto de Investigación en Recursos Cinegéticos (IREC), Consejo Superior de Investigaciones Científicas (CSIC), Universidad de Castilla-La Mancha (UCLM)-Junta de Comunidades de Castilla-La Mancha (JCCM), Ronda de Toledo s/n, 13005 Ciudad Real, Spain; kpauldavis@gmail.com (P.D.K.); marinelacr@hotmail.com (M.C.); 2 National Livestock Resources Research Institute (NaLIRRI/NARO), P.O. Box 5704 Kampala, Uganda; freddykabi@gmail.com (F.K.); mugerwaswidiq@gmail.com (S.M.); 3 Interdisciplinary Laboratory of Clinical Analysis, Interlab-UMU, Regional Campus of International Excellence Campus Mare Nostrum, University of Murcia, Espinardo, 30100 Murcia, Spain; 4 Department of Veterinary Pathobiology, Center for Veterinary Health Sciences, Oklahoma State University, Stillwater, OK 74078, USA

**Keywords:** cattle, tick, vaccine, subolesin, akirin, uganda

## Abstract

Cattle tick infestations and transmitted pathogens affect animal health, production and welfare with an impact on cattle industry in tropical and subtropical countries. Anti-tick vaccines constitute an effective and sustainable alternative to the traditional methods for the control of tick infestations. Subolesin (SUB)-based vaccines have shown efficacy for the control of multiple tick species, but several factors affect the development of new and more effective vaccines for the control of tick infestations. To address this challenge, herein we used a regional and host/tick species driven approach for vaccine design and implementation. The objective of the study was to develop SUB-based vaccines for the control of the most important tick species (*Rhipicephalus appendiculatus*, *R. decoloratus* and *Amblyomma variegatum*) affecting production of common cattle breeds (*Bos indicus* and *B. indicus* x *B. taurus* crossbred) in Uganda. In this way, we addressed the development of anti-tick vaccines as an intervention to prevent the economic losses caused by ticks and tick-borne diseases in the cattle industry in Uganda. The results showed the possibility of using SUB antigens for the control of multiple tick species in *B. indicus* and crossbred cattle and suggested the use of *R. appendiculatus* SUB to continue research on vaccine design and formulation for the control of cattle ticks in Uganda. Future directions would include quantum vaccinology approaches based on the characterization of the SUB protective epitopes, modeling of the vaccine E under Ugandan ecological and epidemiological conditions and optimization of vaccine formulation including the possibility of oral administration.

## 1. Introduction

Cattle tick ectoparasites affect animal health, production and welfare particularly in tropical and subtropical countries of the world [1,2,3]. In particular in Uganda where this study was conducted, tick-borne diseases (TBD) such as East Coast fever (caused by *Theileria parva*), babesiosis (caused by *Babesia bigemina*), anaplasmosis (caused by *Anaplasma marginale*) and heartwater (caused by *Ehrlichia ruminantium*) affect cattle production with estimated losses of over USD 1.1 billion annually [4].

Traditional methods for the control of tick infestations and TBD have been based on acaricides, repellents, antibiotics, cattle breeding and extension education about recommended practices to reduce exposure to ticks [5,6,7]. However, these practices have been only partially successful and drug resistance and contamination impact on public and environmental health constitute important limitations [6,8]. Taken together, these facts encourage the development of vaccines as effective and environmentally sound control strategies for the integrated control of tick infestations and TBD [6,7,9,10,11].

The first and only vaccines against ectoparasites were registered in the early 1990s for the control of cattle tick infestations [9]. These vaccines based on the *Rhipicephalus microplus* midgut concealed antigen BM86 proved to reduce cattle tick populations and the use of acaricides when applied over time for cattle vaccination [9,12,13]. Recently, research has advanced the identification and characterization of tick protective antigens such as subolesin (SUB) [13,14], but several factors affect the development of new and more effective vaccines for the control of tick infestations [7,15].

One of the major limitations for developing effective vaccines for the control of tick infestations and tick-borne pathogens is the lack of funding and the need to fulfil the regulatory requirements for vaccine registration. To address this challenge, we support the use of regional and host/tick species driven approaches for vaccine design and implementation [4,16].

In the study reported here and based on the collaboration between the Spanish Instituto de Investigación en Recursos Cinegéticos (IREC) and the National Agricultural Research Organization of Uganda (NARO) [4], we focused on the development of a SUB-based vaccine for the control of the most important tick species (*Rhipicephalus appendiculatus*, *R. decoloratus* and *Amblyomma variegatum*) affecting production of common cattle breeds (*Bos indicus* and *B. indicus* x *B. taurus* crossbred) in Uganda. SUB, the ortholog of akirin (AKR) in ticks was chosen as a vaccine antigen because it is highly conserved, both genetically and functionally, across all tick species and has shown protection for the control of tick infestations and pathogen infection and transmission [17]. In this way, we addressed the development of anti-tick vaccines as one of the recently proposed measures by the Food and Agriculture Organization of the United Nations (FAO) in conjunction with the Government of Uganda [4] to prevent the economic losses caused by ticks and TBD in the cattle industry in Uganda. The results showed the possibility of using SUB antigens for the control of multiple tick species in *B. indicus* and crossbred cattle and suggested the use of *R. appendiculatus* SUB to continue research on vaccine design and formulation for the control of cattle ticks in Uganda.

## 2. Materials and Methods

### 2.1. Ethics Statement

The experimental cattle were treated in accordance to the Uganda National Council of Science and Technology (UNCT) guiding Principles for Biomedical Research Involving Animals. The experiments were conducted under approval of NARO Institutional Animal Care and Use Committee (IACUC) (No. 2020-0802-20).

### 2.2. Cattle

*Bos indicus* and *B. indicus* × *B. taurus* crossbred cattle breeds were included in the vaccination trials. Cattle had no previous exposure to ticks. Before the experiment, the general health status of the animals was assessed with particular attention to theileriosis and babesiosis. Blood samples were collected and screened both microscopically and by PCR for the identification of piroplasms and *Anaplasma marginale*. PCR was conducted with oligonucleotide primers for *Babesia* and *Theileria* spp. BAB GF2 (forward: 5′-GTC TTG TAA TTG GAA TGA TGG-3′ and reverse: 5′-CCA AAG ACT TTG ATT TCT CTC-3′) and *Anaplasma* spp. 16SANA (forward: 5′-CAGAGTTTGATCCTGGCTCAGAACG-3′ and reverse: 5′-GAGTTTGCCGGGACTTCTTCTGTA-3′) as previously described [18,19]. Healthy *B. indicus* (*n* = 20) and crossbred (*n* = 20) calves aged at least ten months were selected for the study. The animals were housed individually in arthropod-free, well ventilated isolation pens and fed on fodder and 20% protein concentrate receiving water ad libitum.

### 2.3. Ticks

Ticks *R. appendiculatus*, *A. variegatum* and *R. decoloratus* were originally collected from different agroecological zones of Uganda in 2012 and subsequently maintained by feeding under controlled conditions on calves and rabbits in the NaLIRRI tick colony. Ticks were screened by PCR as described for cattle and were negative for tick-borne pathogens *Babesia*, *Theileria* and *Anaplasma* spp. eggs oviposited by various ticks of the same species were pooled and hatched larvae were first kept for 15 days in humidity chambers at 12h light:12h dark photoperiod, 20 °C and 95% relative humidity before application on to the animals.

### 2.4. Cloning of SUB-Coding Genes and Sequence Analysis

The genes coding for SUB were cloned from adult female *R. appendiculatus*, *A. variegatum* and *R. decoloratus* tick tissues. Total RNA was extracted from midguts of the three tick species using TRI Reagent (Sigma-Aldrich, St. Louis, MO, USA) following the manufacturer’s instructions. The RNA was used for cDNA synthesis using the iScript cDNA synthesis Kit (Bio-Rad Laboratories Inc., Hercules, CA, USA) followed by *sub* gene amplification by PCR using gene-specific forward (F) and reverse (R) oligonucleotide primers for each tick species (*R. appendiculatus*, F: 5′-CCAATGGCTTGTGCGACATTAAA-3′, R: 5′-CGACAAATAGCTGGGCGTA-3′; *A. variegatum*, F: 5′-CACCATGGCTTGCGCAACATTAAA-3′, R: 5′-TTTGGTCGTACGTAAACTTG-3′; *R. decoloratus*, F: 5′-CACCATGGCTTGCGCAACATTAAA-3′, R: 5′-TTTGGTCGTACGTAAACTTGAC-3′). SUB sequences were deposited in the GenBank (Accession numbers MT241514, MT241515, MT252964). For SUB amino acid sequence analysis, entries for *Rhipicephalus* spp. complete sequences (*n* = 31) and *Amblyomma* spp. partial and complete sequences (*n* = 6) available on GenBank (https://www.ncbi.nlm.nih.gov/protein; accessed 20 March 2020) were used together with sequences from Ugandan tick strains for a total of 33 and 7 sequences for *Rhipicephalus* and *Amblyomma* spp., respectively. *Ixodes scapularis* SUB (AAV67031.1) was used as the outgroup. Sequences were aligned using the COBALT constraint-based multiple alignment tool (https://www.ncbi.nlm.nih.gov/tools/cobalt/cobalt.cgi?LINK_LOC=BlastHomeLink). The evolutionary analysis was performed using the maximum likelihood method and Jones-Taylor-Thornton (JTT) matrix-based model [20]. Trees with the highest log likelihood (−1075.01 and −722.52 for *Rhipicephalus* and *Amblyomma* spp., respectively) were used. Initial trees for the heuristic search were obtained automatically by applying neighbor-joining (NJ) and BioNJ algorithms to a matrix of pairwise distances estimated using a JTT model, and then selecting the topology with superior log likelihood value. For *Rhipicephalus* spp., a discrete Gamma distribution was used to model evolutionary rate differences among sites (five categories; +G, parameter = 1.7321). Evolutionary analyses were conducted using MEGA X [21].

### 2.5. Production of Recombinant SUB Antigens and Vaccine Formulation

For the production of recombinant SUB proteins, *Escherichia coli* BL21 Star (DE3) One Shot cells (Invitrogen-Life Technologies, Inc., Grand Island, NY, USA) were transformed with the target *sub* gene cloned into the pET101/D-TOPO expression vector (Ref. K101-01, Invitrogen-Life Technologies). The cells were cultured in 10 mL Luria–Bertani (LB) medium containing 50 μg/mL ampicillin (Sigma-Aldrich) and 0.5% glucose (Laboratorios CONDA S.A., Madrid, Spain) overnight at 37 °C with shaking. Cells from 9 mL overnight culture were inoculated into 1000 mL flasks containing 200 mL LB with 50 μg/mL ampicillin and 0.5% glucose and incubated at 37 °C and shaking at 200 rpm for 2 h followed by 4–6 h after addition of 0.5 mM final concentration of isopropyl-β-dthiogalactopyranoside (IPTG, Sigma-Aldrich) for induction of gene expression for SUB production. The cells were harvested by centrifugation at 3900× *g* for 15 min at 4 °C and the cell pellet stored at −80 °C until purification.

One gram cells were resuspended in 5 mL of lysis buffer (50 mM K_3_PO_4_, 400 mM NaCl, 100 mM KCl, 7M Urea, 10 mM imidazole, pH 7.8) containing a protease inhibitor (Ref. 04693132001, Roche, San Cugat del Vallés, Barcelona, Spain) and disrupted using a cell sonicator (Model MS73; Bandelin Sonopuls, Berlin, Germany). After disruption, the insoluble protein fraction containing the antigens as inclusion bodies were collected in the supernatant by centrifugation at 10,000× *g* for 15 min at 4 °C and stored at −20 °C. Recombinant proteins were purified by Ni affinity chromatography using 1 mL HisTrap FF columns mounted on the AKTA^TM^ fast protein liquid chromatography (AKTA-FPLC) system (GE Healthcare, Piscataway, NJ, USA) in the presence of 7M urea. The eluted fraction containing the purified proteins was dialyzed overnight against 1000 volumes of PBS (137 mM NaCl, 2.7 mM KCl, 10 mM Na_2_HPO_4_, 1.8 mM KH_2_PO_4_) pH 7.4 for 12 h at 4 °C. Protein concentration was determined using bicinchoninic acid (Ref. 23225, Pierce BCA Protein Assay Kit, Thermo Scientific, Rockford, IL, USA).

Recombinant proteins were adjuvated in Montanide ISA 50 V2 (Seppic, Paris, France) in a stable water in oil (W/O) vaccine emulsion at a concentration of 50 µg SUB per ml and stored at 4 °C [22].

### 2.6. Western Blot Analysis of Recombinant SUB Antigens

Ten μg per well of purified recombinant proteins were loaded onto an SDS-12% polyacrylamide gel (Life Science, Hercules, CA, USA). Gels were either stained with Coomassie Brilliant Blue or used for Western blot analysis. For Western blot analysis, the gel was transferred to a nitrocellulose membrane which was then blocked with 5% BSA (Sigma-Aldrich) for 2 h at RT, washed four times with Tris-buffered saline (TBS; 50 mM Tris-Cl, pH 7.5, 150 mM NaCl, 0.5% Tween 20) and incubated with pooled sera collected from vaccinated cattle at day 60. Sera with primary antibodies were used at a 1:300 dilution in TBS, and the membrane was incubated overnight at 4 °C and washed four times with TBS. The membrane was then incubated with an anti-bovine IgG-horseradish peroxidase (HRP) conjugate (Sigma-Aldrich) diluted 1:5000 in TBS with 3% BSA (BSA/TBS). The membrane was washed five times with TBS and finally developed with 3,3′, 5,5′-tetramethylbenzidine (TMB) stabilized substrate for HRP (Promega, Madrid, Spain) according to the manufacturer recommendations. Molecular weight markers (Spectra multicolor broad range protein ladder; Thermo Scientific) were used.

### 2.7. Vaccination Trials

The vaccination trials were conducted using a double blinded design, i.e., each of the vaccine formulations was coded as S1, S2, S3, S4 and S5 such that neither researchers involved in vaccination and data collection or in data analysis knew which vial contains what vaccine formulation until the completion of the trial. Calves of each breed were randomly assigned to five groups of four animals each. Calves in each group were vaccinated with vaccine formulations containing each of the SUB antigens, a cocktail of all three SUB antigens or adjuvant alone as control. The animals were injected intramuscularly in the neck muscles with 2 mL vaccine (100 µg SUB per dose) on days 0, 30 and 60. Fifteen days after the last immunization (day 75), crossbred cattle were challenged with *R. appendiculatus, R. decoloratus* and *A. variegatum* tick larvae, simultaneously. The three tick infestation treatments were done on each animal using two ear-bags for *R. appendiculatus* and *A. variegatum* and a cell glued on the back of the animal for *R. decoloratus*. *B. indicus* cattle were infested with *R. appendiculatus* and *A. variegatum* tick larvae simultaneously as described for crossbred calves. Infestation with *R. decoloratus* was not included in *B. indicus* due to the limited number of available larvae and the highest resistance to one-host tick species in this cattle breed [23]. All data was collected and analyzed from each individual calve.

For the three host tick species, *R. appendiculatus* and *A. variegatum*, approximately 300 larvae were applied on each animal. The larvae were allowed to attach and engorge for two weeks. The ear bags were then removed, and successfully engorged ticks counted, weighed and incubated for molting at 20 °C and 95% relative humidity. A 15-day-old batch of 200 nymphs were applied on to each ear for two weeks and engorged nymphs were collected, counted, weighed and incubated for molting to adults. Finally, 30 starved adult male and female ticks were applied on to the calf’s ear at a one male per three female ratio in the ear-bags. After two weeks, the ear-bags were removed and engorged female ticks collected, counted, weighed individually and incubated for oviposition. Eggs laid per tick were weighed for each calf and incubated for hatching. Larvae obtained from each egg batch were weighed.

For the one host tick, *R. decoloratus*, approximately 300 larvae were applied on to each calf using a cell glued to the back of the animal. The cells were left in position for 28–30 days to allow all the developmental stages to take place on the host. The cells were then removed, and adult engorged ticks collected, counted, weighed individually and incubated for oviposition. The eggs mass per female tick was weighed and incubated for hatching. The recovered larvae per egg batch were weighed.

### 2.8. Data Collection and Analysis of Vaccine Efficacy

The effect of the vaccination on tick life cycle was determined using the formulae previously described [13,24,25].

Effect on the number of engorged larvae (DL), nymphs (DN) and adult female ticks (DA):

DL, DN, DA (%) = 100 (1 − NTV/NTC)(1)

where DL, DN, DA (%) is the percentage reduction of ticks, NTV is the number of ticks dropping off the animals in the vaccinated group and NTC is the number of ticks dropping off the animals in the control group.

Effect on the molting of tick larvae (DMn) and nymphs (DMa):

DMn, DMa (%) = 100 (1 − MTV/MTC)(2)

where DMn, DMa (%) is the percentage reduction of average molting of ticks, MTV is the average molting of ticks in the vaccinated group and MTC is the average molting of ticks in the control group

Effect on the tick oviposition (DO):

DO (%) = 100 (1 − PATV/PATC)(3)

where DO (%) is the percentage reduction in tick oviposition, PATV is the average weight of the eggs per survived tick in the vaccinated group and PATC is the average weight of the eggs per survived tick in the control group.

Effect on fertility (DF):

DF (%) = 100 (1 − PPLOV/PPLOC)(4)

where DF (%) is the percentage reduction in egg fertility, PPLOV is the average weight of the larvae per gram of eggs in the vaccinated group and PPLOC is the average weight of the larvae per gram of eggs in the control group.

Vaccine efficacy (E):

E (%) = 100 (1 − (DL × DMn × DN × DMa × DA × DO × DF)).(5)

Results were included in Supplementary Materials Data S1. Data were analyzed statistically to compare results for each tick species between individuals fed on vaccinated and adjuvant/saline injected control cattle by Chi-square test (*p* = 0.05; *n* = 4 biological replicates). Only parameters with statistically significant differences were included in the vaccine E calculation. Total vaccine E for each antigen in all tick species was compared between *B. indicus* and crossbred cattle by a Student’s t-test with unequal variance (*p* = 0.05; *n* = 2–3, i.e., three tick species in crossbred cattle or two tick species in *B. indicus*). A Spearman’s Rho correlation analysis (https://www.socscistatistics.com/tests/spearman/Default2.aspx) was performed between total vaccine E values for each antigen in all cattle breeds and tick species (*p* = 0.05; *n* = 5, i.e., three tick species in crossbred cattle plus two tick species in *B. indicus*).

### 2.9. Characterization of the Antibody Response in Vaccinated Calves

Blood samples were collected before each vaccination (days 0, 30 and 60), at day 45 between second and third vaccinations and at the end of the experiment (days 180 or 195 for *B. indicus* and crossbred cattle, respectively). The obtained serum was stored at −20 °C until analysis. Serum IgG antibody titers were determined using an indirect antigen-specific ELISA [25,26]. Purified recombinant SUB antigens (0.1 µg in 50 µL of carbonate-bicarbonate buffer; Sigma-Aldrich) per well were used for overnight coating of high absorption capacity polystyrene microtiter ELISA plates at 4 °C. Plates were blocked with 200 µL/well of blocking solution (10% fetal bovine serum in PBS, 137 mM NaCl, 2.7 mM KCl, 10 mM Na2HPO4, 1.8 mM KH2PO4 and pH 7.4) (Sigma-Aldrich). The sera were serially diluted to 1:10, 1:100, 1:1000 and 1:10,000 v/v in blocking solution (optimal dilution, 1:1000). Plates were then incubated with 50 µL/well of diluted sera overnight at 4 °C, followed by three washes using PBS and 0.1% Tween 20 (PBST) and an incubation with PBS-diluted (1:10,000) rabbit anti-bovine IgG-HRP conjugates (Sigma-Aldrich) for 1 h at room temperature (RT). After three washes with PBST, the chromogenic reaction was developed with 3,3′5,5′-tetramethylbenzidine (Sigma-Aldrich), stopped with 50 µL/well of 3N H_2_SO_4_ and the optical density at 450 nm (O.D._450 nm_) was determined in an ELISA plate reader. Antibody titers were expressed as the O.D._450 nm_ values and compared between vaccinated and control groups using a one-way ANOVA test (https://www.socscistatistics.com/tests/anova/default2.aspx) (*p* = 0.05; *n* = 4 biological replicates). In addition, a correlation analysis was performed in Microsoft Excel between the values of the parameters of *Rhipicephalus* spp. tick life stages used for vaccine E calculation that showed significant differences with at least three of the SUB vaccine formulations and the anti-SUB antibody titers against *R. appendiculatus* SUB at day 60 before tick challenge in individual animals. Data was analyzed using a Spearman’s Rho correlation analysis (https://www.socscistatistics.com/tests/spearman/Default2.aspx) (*p* = 0.05; *n* = 20, i.e., 4 animals for each of the 5 vaccine formulations including the control).

## 3. Results and Discussion

### 3.1. Experimental Design and Rationale

**Figure 1:**
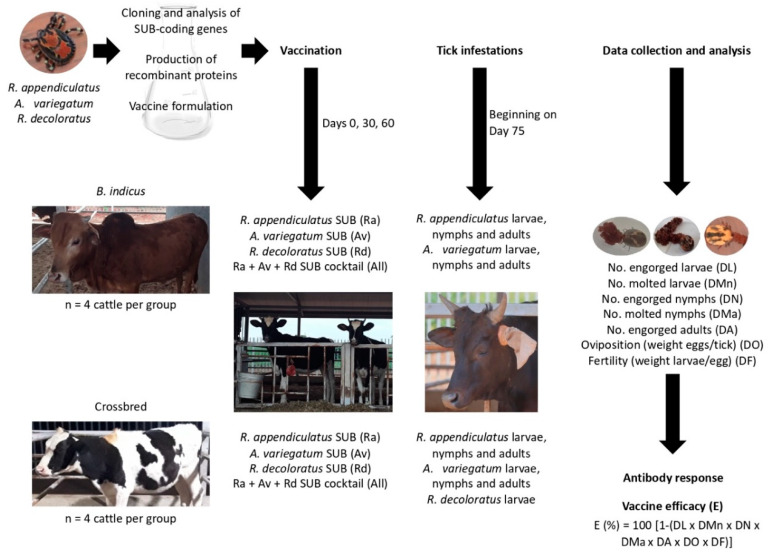
Experimental design. The study design included the cloning and analysis of subolesin (SUB)-coding genes in Ugandan strains of *R. appendiculatus*, *R. decoloratus* and *A. variegatum*, followed by production of recombinant proteins and vaccine formulations with single and all combined antigens. Vaccination trials were conducted in the most common cattle breeds (*Bos indicus* and *B. indicus* × *B. taurus* crossbred) in Uganda using 4 calves/group and infested with *R. appendiculatus* and *A. variegatum* larvae, nymphs and adults and *R. decoloratus* larvae in crossbred cattle only. The effect of vaccination on cattle antibody response and on different tick developmental stages (number of engorged larvae (DL), nymphs (DN) and adult female ticks (DA), molting of tick larvae (DMn) and nymphs (DMa), oviposition (DO) and fertility (DF) was used to evaluate vaccine E.

The study was designed to approach the objective of evaluating the protective capacity of recombinant SUB-based vaccines against multiple tick species infesting cattle in Uganda (Figure 1). The experimental design included the cloning and analysis of SUB-coding genes in Ugandan strains of *R. appendiculatus*, *R. decoloratus* and *A. variegatum*, and production of recombinant proteins and vaccine formulations with single and all combined antigens. SUB, the ortholog of AKR in ticks was chosen as a vaccine antigen because it is highly conserved both genetically and functionally across all tick species and has shown protection in cattle for the control of tick infestations and pathogen infection and transmission [17,27,28]. Vaccination trials were conducted in the most common cattle breeds (*Bos indicus* and *B. indicus* × *B. taurus* crossbred) in Uganda using four calves per group which were infested with *R. appendiculatus* and *A. variegatum* larvae, nymphs and adults. Due to the limited number of available larvae and the high resistance of *B. indicus* cattle to one-host tick species [23], only crossbred cattle were infested with *R. decoloratus* larvae. The effect of vaccination on cattle antibody response and on different tick developmental stages (DL, DMn, DN, DMa, DA, DO and DF) was used to evaluate vaccine E. Only parameters with statistically significant differences were included in the vaccine E calculation.

### 3.2. SUB Protein Sequences Are Highly Conserved but Show Distinctive Amino Acid Residues

**Figure 2:**
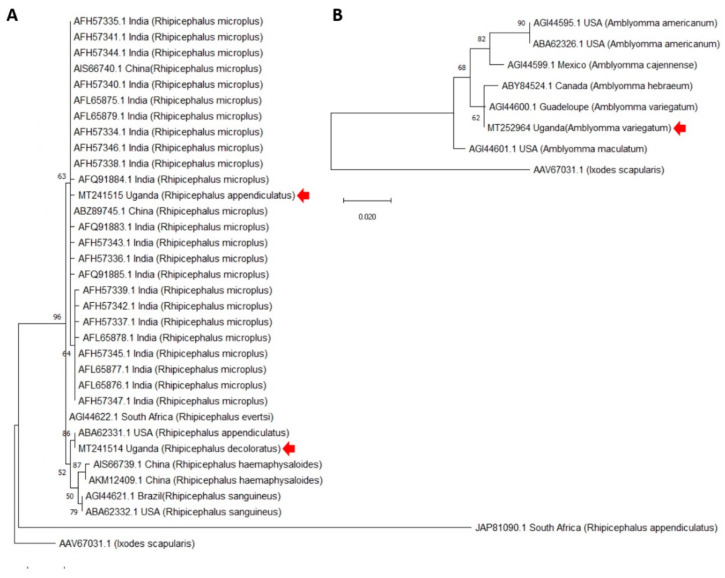
Phylogenetic analysis of SUB protein sequences. The evolutionary analysis was performed using the maximum likelihood method and JTT matrix-based model in Mega X. (**A**) *Rhipicephalus* spp.: The tree with the highest log likelihood (−1075.01) is shown. The percentage of trees in which the associated taxa clustered together is shown next to the branches. A discrete Gamma distribution was used to model evolutionary rate differences among sites (5 categories; +G, parameter = 1.7321). The tree is drawn to scale, with branch lengths measured in the number of substitutions per site. This analysis involved 34 amino acid sequences with a total of 192 positions in the final dataset. (**B**) *Amblyomma* spp.: The tree with the highest log likelihood (−722.52) is shown. The percentage of trees in which the associated taxa clustered together is shown next to the branches. The tree is drawn to scale, with branch lengths measured in the number of substitutions per site. This analysis involved 8 amino acid sequences with a total of 184 positions in the final dataset. Sequences from Ugandan tick strains are marked with a red arrow.

**Figure 3:**
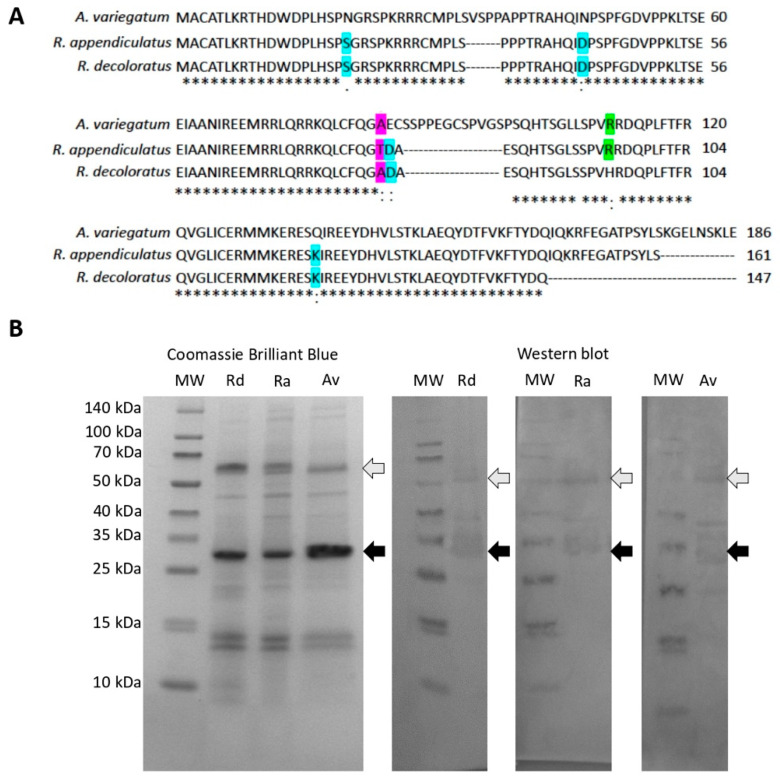
Production of recombinant *R. appendiculatus*, *R. decoloratus* and *A. variegatum* SUB in *E. coli*. (**A**) alignment of SUB amino acid protein sequences. Distinctive amino acid residues are highlighted (blue: Identical only between *R. appendiculatus* and *R. decolortus*, green: Identical only between *A. variegatum* and *R. appendiculatus* and pink: Identical only between *A. variegatum* and *R. decoloratus*). Conserved residues between all sequences are indicated with asterisks (*). (**B**) ten μg per well of purified recombinant proteins were loaded into an SDS-12% polyacrylamide gel. Gels were stained with Coomassie Brilliant Blue or used for Western blot analysis. For Western blot analysis, the gel was transferred to a nitrocellulose membrane and the membrane was incubated with pooled sera collected from vaccinated cattle at day 60. The positions of the monomer and dimer recombinant proteins is indicated with black and grey arrows, respectively. Abbreviation: MW, molecular weight markers (Spectra multicolor broad range protein ladder; Thermo Scientific).

The SUB-coding genes were cloned from *R. appendiculatus*, *R. decoloratus* and *A. variegatum* Ugandan tick strains. In accordance with previous results [29,30,31], protein sequence analysis showed high sequence homology (Figure 2A,B). The Ugandan *R. appendiculatus* sequence clustered together with *R. microplus* from Asia while Ugandan *R. decoloratus* was closely related to a *R. appendiculatus* from USA (Figure 2A). With a limited number of sequences available, Ugandan *A. variegatum* clustered together with *A. variegatum* and *A. hebraeum* from America (Figure 2B). However, when focusing on Ugandan tick species, SUB protein sequences showed a high homology (75% amino acid identity) but with some distinctive amino acid residues (Figure 3A). Recombinant proteins were produced in *E. coli* with the expected molecular weight (29 and 30 kDa for *Rhipicephalus* spp. and *A. variegatum*, respectively) for both monomer and dimer forms, which are commonly found in SUB/AKR with functional implications [32] (Figure 3B).

### 3.3. The Antibody Response Was Higher in All Vaccinated Cattle when Compared to Adjuvant-Alone Treated Controls

**Figure 4:**
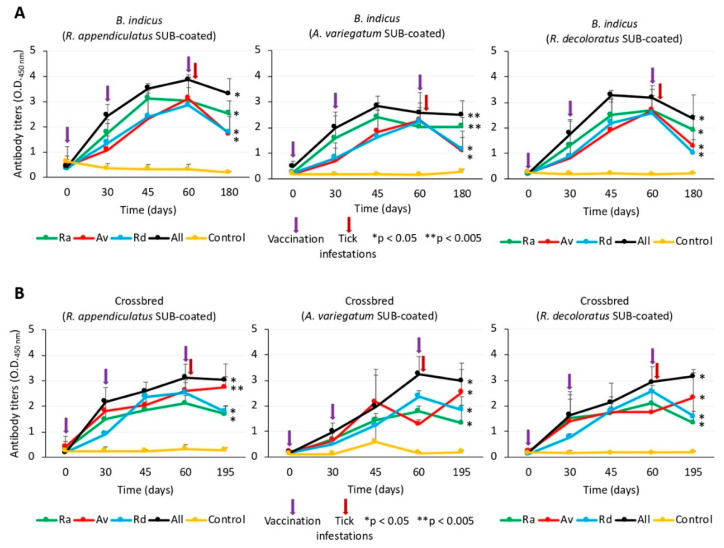
Antibody response in SUB-vaccinated and control cattle. (**A**) *B. indicus* cattle. (**B**) crossbred cattle. Blood samples were collected before each vaccination (days 0, 30 and 60; violet arrows), at day 45 between second and third vaccinations and at the end of the experiment (days 180 or 195 for *B. indicus* and crossbred cattle, respectively). Serum IgG antibody titers were determined using an indirect antigen-specific ELISA. Antibody titers were expressed as the OD_450 nm_ values and compared between vaccinated and control groups using a one-way ANOVA test (https://www.socscistatistics.com/tests/anova/default2.aspx) (* *p* < 0.05, ** *p* < 0.005; *n* = 4 biological replicates).

Antibody response to vaccination with SUB has been shown to be the main protective mechanism against tick infestations [33], although cell-mediated immunity may play a role with certain vaccine formulations [34]. Anti-SUB antigen-specific IgG antibody titers were determined on each vaccinated and adjuvant-alone treated controls in sera collected before each vaccination (days 0, 30 and 60), at day 45 between second and third vaccinations and at the end of the experiment (days 180 or 195 for *B. indicus* and crossbred cattle, respectively) (Figure 4A,B). The results showed that antibody titers increased after the first and second vaccination doses but in some cases decreased after the third vaccination dose (Figure 4A,B). These results may reflect antigen-specific and dose effects that have been previously shown in SUB-based vaccines [35,36]. These effects may affect vaccine efficacy and require optimization [37,38,39]. Nevertheless, the anti-SUB antibody titers were significantly (* *p* < 0.05 and * *p* < 0.005) higher throughout the experiment in vaccinated cattle when compared to controls (Figure 4A,B).

### 3.4. Vaccination Affected Multiple Tick Developmental Stages with Differences between SUB Antigens, Tick Species and Cattle Breeds

**Figure 5:**
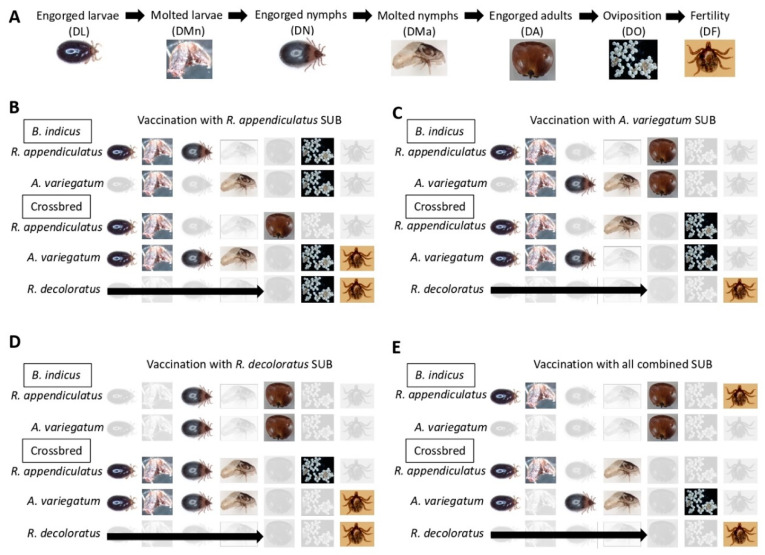
Effect of vaccination with SUB on different tick developmental stages. (**A**) tick developmental stages included in the analysis. (**B**) vaccination with *R. appendiculatus* SUB. (**C**) vaccination with *A. variegatum* SUB. (**D**) vaccination with *R. decoloratus* SUB. (**E**) vaccination with all combined SUB formulation. The tick developmental stages included DL, DN, DA, DMn, DMa, DO and DF. Only parameters with statistically significant differences (Chi-square test; *p* < 0.05, *n* = 4 biological replicates; Data S1) are shown here and were included in the vaccine E calculation.

The results of the vaccination showed differences between SUB antigens, tick species and cattle breeds (Data S1, Figure S1A–E, summarized in Figure 5A–E and Table 1). The total number of tick developmental stages included in the analysis corresponded to seven (DL, DMn, DN, DMa, DA, DO, DF) for three-host ticks and three (DA, DO, DF) for the one-host tick, *R. decoloratus* (Figure 5A). Only parameters with statistically significant differences (*p* < 0.05) were included in the analysis. At the SUB antigen level and considering the total number of tick developmental stages that could be affected in all groups (*n* = 31; Figure 5B–E), the number of tick developmental stages affected by vaccination varied from 18/31 (58%) for *R. appendiculatus* SUB to 16/31 (52%) for *A. variegatum* SUB, 15/31 (48%) for *R. decoloratus* SUB and 13/31 (42%) for all combined SUB antigens. At the tick species level and considering the total number of developmental stages affected after vaccination in all groups (*n* = 56 for *R. appendiculatus* or *A. variegatum* and *n* = 12 for *R. decoloratus*; Figure 5B–E), the results were similar for *R. appendiculatus* (28/56, 50%), *A. variegatum* (28/56, 50%) and *R. decoloratus* (5/12, 42%). When comparing the results between the cattle breeds in all groups, the total number of tick developmental stages that could be affected in *B. indicus* were 56 for the two three-host tick species and in crossbred cattle were 68 for all three tick species (Figure 5B–E). In this case, the results showed 23/56 (41%) and 39/68 (57%) affected tick developmental stages in *B. indicus* and crossbred cattle, respectively.

One consideration that may be relevant for evaluating the effect of vaccination on the control of three-host tick infestations is the effect on reducing early tick developmental stages (i.e., DL and DMn, *n* = 8 for each SUB vaccine antigen; Figure 5B–E). Focusing on these parameters, the results showed that 7/8 (88%), 7/8 (88%), 4/8 (50%) and 5/8 (63%) of the early tick developmental stages were reduced by vaccination with *R. appendiculatus* SUB, *A. variegatum* SUB, *R. decoloratus* SUB and all combined SUB antigens, respectively.

In summary, these results showed that vaccination with SUB affected tick developmental stages with minor differences between tick species and a higher overall effect of vaccination in crossbred cattle considering all antigen formulations. As found in this study, minor differences between tick species in the efficacy of vaccination with SUB has been reported before [33]. Furthermore, vaccination with *R. appendiculatus* SUB affected the highest number of developmental stages in all tick species with a high reduction of early developmental stages in three-host ticks.

### 3.5. The Results of Vaccine E Support the Possibility of Using SUB Antigens for the Control of Multiple Tick Species in Different Cattle Breeds

**Figure 6:**
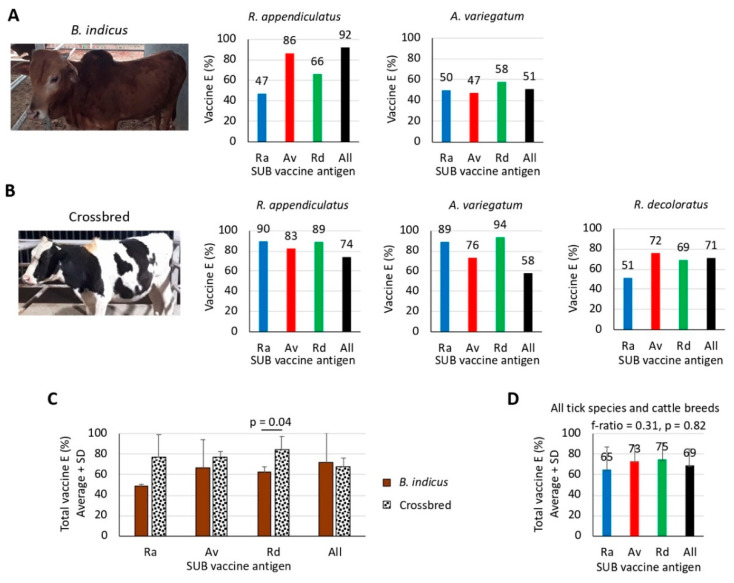
Analysis of vaccine E. Vaccine E (%) was calculated as 100 (1−(DL × DMn × DN × DMa × DA × DO × DF)), and only parameters with statistically significant differences (Chi-square test; *p* < 0.05, *n* = 4 biological replicates; Data S1) were included in the analysis. (**A**) results of the vaccination trials in *B. indicus* cattle. (**B**) results of the vaccination trials in crossbred cattle. (**C**) total vaccine E for each antigen against all tick species was compared between *B. indicus* and crossbred cattle by a Student’s t-test with unequal variance (*p* = 0.05; *n* = 2–3, i.e., three tick species in crossbred cattle or two tick species in *B. indicus*). (**D**) a Spearman’s Rho correlation analysis was performed between total vaccine E values for each antigen in all cattle breeds and tick species (f-ratio = 0.31, *p* = 0.82; *n* = 5, i.e., three tick species in crossbred cattle plus two tick species in *B. indicus*).

The results of the effect of vaccination on the different tick developmental stages translates into vaccine E (Figure 6A,B). However, in this case the number of affected tick developmental stages is not as relevant as the percentage by which these developmental stages were reduced (Table 1). The vaccine E varied from 47% to 94% between groups, but in most cases, it was higher than 50% (Figure 6A,B), which was the limit originally set for BM86-based anti-tick vaccines [40]. Except for vaccine E with *R. appendiculatus* SUB against infestations by *R. appendiculatus* in crossbred cattle (Figure 6B), vaccine E was not the highest when the SUB antigen was of the same origin as the infestation tick species (Figure 6A,B). Furthermore, except for *R. appendiculatus* infestations of *B. indicus* cattle, vaccine E was not higher with all combined SUB antigens when compared to individual antigens (Figure 6A,B). It has been previously shown that differences in protein metabolism between tick species and immunologic interference with antigen combinations may affect tick vaccine efficacy [41,42]. These facts may at least partially explain why vaccine E for homologous pairs infesting tick-SUB antigen was generally lower than heterologous pairs or why vaccine E for combined SUB antigen formulations was lower than for individual antigens. As shown above with tick developmental stages, vaccine E tended to be higher in crossbred than in *B. indicus* cattle with significant differences (*p* = 0.04) for *R. decoloratus* SUB only (Figure 6C). Integrating the results from all tick species and cattle breeds, the total vaccine E varied from 65% to 75% but without significant differences (*p* = 0.82) between SUB antigens (Figure 6D).

**Figure 7:**
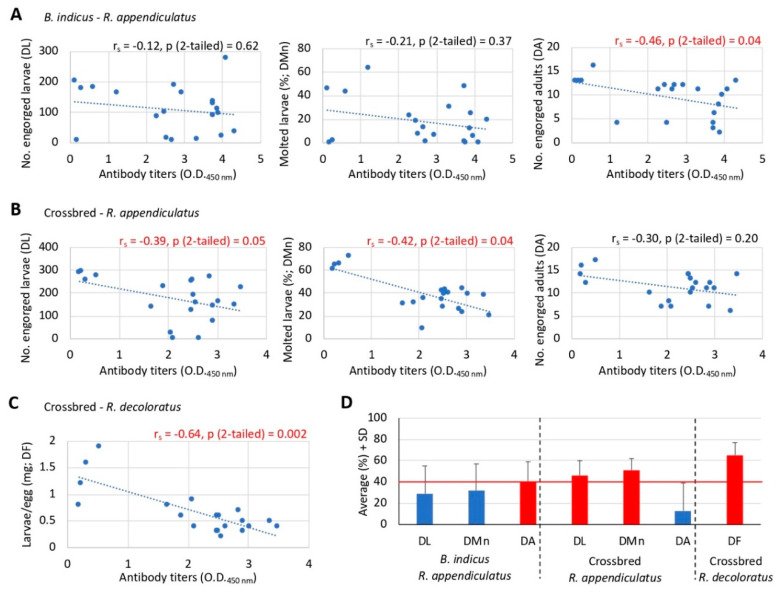
Correlation analysis between anti-SUB antibody titers and vaccination effect on different tick developmental stages. (**A**) *B. indicus* cattle infested with *R. appendiculatus*. (**B**) crossbred cattle infested with *R. appendiculatus*. (**C**) crossbred cattle infested with *R. decoloratus*. (**D**) values (average + SD) of the parameters of the tick developmental stages used in the analysis (in red are shown the parameters with significant negative correlation with anti-SUB antibody titers and the threshold value line for observing significant differences). The correlation analyses were conducted between the values of the parameters of *Rhipicephalus* spp. tick life stages used for the calculation of vaccine E that showed significant differences with at least three of the SUB vaccine formulations (Table 1) and the anti-*R. appendiculatus* SUB antibody titers at day 60 before tick challenge. All animals in both vaccinated and control groups were included in the analysis. Data was analyzed using a Spearman’s Rho correlation analysis (*p* ≤ 0.05; *n* = 20, i.e., 4 animals for each of the 5 vaccine formulations including the control).

The IgG antibody response is an important correlate of protection in anti-tick vaccines [13,28,35,36,43,44,45,46]. Therefore, to explore this concept herein we conducted correlation analyses between the values of the parameters of *Rhipicephalus* spp. tick life stages used for the calculation of vaccine E that showed significant differences with at least three of the SUB vaccine formulations (Figure 6B,C and Table 1) and the anti-*R. appendiculatus* SUB antibody titers at day 60 before tick challenge (Figure 7A–C). All animals in both vaccinated and control groups were included in the analysis.

As expected from previous experiments [28,35,36,43,44,45,46], a tendency towards a negative correlation between antibody titers and tick life stage parameters was observed (Figure 7A–C). The results showed that for *R. appendiculatus* ticks, a significant negative correlation (*p* ≤ 0.05) was observed for DA in *B. indicus* (Figure 7A) and for DL and DMn in crossbred cattle (Figure 7B). For *R. decoloratus*, a significant negative correlation (*p* = 0.002) was obtained for DF (Figure 7C). Then, using the average values for the parameters of the tick developmental stages used in the analysis (Figure 7A–C), the results showed that a 40% reduction was the threshold value for observing a significant negative correlation between antibody titers and tick life stage parameters (Figure 7D). 

These results support the possibility of using SUB-based vaccines for the control of multiple tick species in different cattle breeds and a role for the antigen-specific antibody response in the reduction of tick infestations after vaccination with SUB.

## 4. Conclusions

The results of the study provided information on SUB genetic diversity among tick species in Uganda with distinctive amino acids. The antigen-specific IgG antibody response to vaccination with SUB was higher throughout the experiment in vaccinated animals than in adjuvant-treated controls. Vaccination with SUB affected various tick developmental stages with minor differences between tick species and a higher vaccine E in crossbred cattle. Cross-protection between the different SUB antigens was observed, reflecting protein sequence homology between SUB from different tick species. Therefore, the results of vaccine E support the possibility of using SUB antigens for the control of multiple tick species in different cattle breeds. Finally, the negative correlation between anti-SUB antibody titers and tick life stage parameters support a role of the antigen-specific antibody response in the reduction of tick infestations after vaccination with SUB.

These results support the possibility of using SUB antigens for the control of multiple tick species in *B. indicus* and crossbred cattle in Uganda. Comparing the different SUB antigen vaccine formulations, the results did not support the use of antigen combination. Instead, vaccination with *R. appendiculatus* SUB affected the highest number of developmental stages in all tick species with a high reduction of early developmental stages in three-host ticks. Furthermore, the vaccine with *R. appendiculatus* SUB was the only one showing a higher E against infestations by tick species with the same origin of the antigen. Based on these evidences, we propose to use *R. appendiculatus* SUB to continue research on vaccine design and formulation. Future directions would include quantum vaccinology approaches based on the characterization of the SUB protective epitopes [32] and tick-pathogen interactions [47], modeling of the vaccine E under Ugandan ecological and epidemiological conditions [4] and optimization of vaccine formulation including the possibility of oral administration to improve cattle welfare and safety [34].

## Figures and Tables

**Table 1:** Results of the SUB vaccination trials.

Vaccination with *R. appendiculatus* SUB								
Group	DL	DMn	DN	DMa	DA	DO	DF	E
*B. indicus*								
*R. appendiculatus*	17% *	22% *	13% *	0%	19%	6% *	0%	47%
*A. variegatum*	0%	25% *	0%	3% *	19%	31% *	0%	50%
**Crossbred**								
*R. appendiculatus*	41% *	64% *	0%	0%	51% *	0%	0%	90%
*A. variegatum*	34% *	44% *	19% *	25% *	0%	4% *	49% *	89%
*R. decoloratus*					0%	8% *	47% *	51%
**Vaccination with *A. variegatum* SUB**								
**Group**	**DL**	**DMn**	**DN**	**DMa**	**DA**	**DO**	**DF**	**E**
***B. indicus***								
*R. appendiculatus*	61% *	51% *	0%	0%	29% *	0%	0%	86%
*A. variegatum*	0%	1% *	7% *	3% *	41%*	0%	0%	47%
**Crossbred**								
*R. appendiculatus*	53% *	39% *	0%	39% *	0%	4% *	0%	83%
*A. variegatum*	54% *	28% *	16% *	0%	0%	14% *	0%	76%
*R. decoloratus*					0%	0%	72% *	72%
**Vaccination with *R. decoloratus* SUB**								
**Group**	**DL**	**DMn**	**DN**	**DMa**	**DA**	**DO**	**DF**	**E**
***B. indicus***								
*R. appendiculatus*	0%	0%	25% *	0%	55% *	0%	0%	66%
*A. variegatum*	0%	0%	32% *	0%	38% *	0%	0%	58%
**Crossbred**								
*R. appendiculatus*	62% *	47% *	25% *	19% *	0%	10% *	0%	89%
*A. variegatum*	69% *	50% *	19% *	18% *	0%	0%	39% *	94%
*R. decoloratus*					0%	0%	69%*	69%
**Vaccination with the combination of SUB antigens**								
**Group**	**DL**	**DMn**	**DN**	**DMa**	**DA**	**DO**	**DF**	**E**
***B. indicus***								
*R. appendiculatus*	38% *	53% *	0%	0%	57% *	0%	38% *	92%
*A. variegatum*	0%	0%	0%	0%	51%*	0%	0%	51%
**Crossbred**								
*R. appendiculatus*	29% *	53% *	0%	22% *	0%	0%	0%	74%
*A. variegatum*	27% *	0%	7% *	28% *	0%	13% *	0%	69%
*R. decoloratus*					0%	0%	71% *	71%

Vaccine E (%) was calculated as 100 (1−(DL × DMn × DN × DMa × DA × DO × DF)), and only parameters with statistically significant differences (Chi-square test; * *p* < 0.05, *n* = 4 biological replicates; Data S1) were included in the analysis.

## Supplementary Materials

The following are available online at https://www.mdpi.com/2076-393X/8/2/319/s1, Figure S1: Effect of vaccination with SUB on different tick developmental stages, Data S1: Results of the vaccination trials.

## References

[1] Jongejan F., Uilenberg G. (2004). The global importance of ticks. Parasitology.

[2] de la Fuente J., Estrada-Peña A., Venzal J.M., Kocan K.M., Sonenshine D.E. (2008). Overview: Ticks as vectors of pathogens that cause disease in humans and animals. Front. Biosci..

[3] Muhanguzi D., Byaruhanga J., Amanyire W., Ndekezi C., Ochwo S., Nkamwesiga J., Mwiine F.N., Tweyongyere R., Fourie J., Madder M. (2020). Invasive cattle ticks in East Africa: Morphological and molecular confirmation of the presence of *Rhipicephalus microplus* in south-eastern Uganda. Parasites Vectors.

[4] de la Fuente J., Contreras M., Kasaija P.D., Gortazar C., Ruiz-Fons J.F., Mateo R., Kabi F. (2019). Towards a multidisciplinary approach to improve cattle health and production in Uganda. Vaccines.

[5] Mapholi N.O., Marufu M.C., Maiwashe A., Banga C.B., Muchenje V., MacNeil M.D., Chimonyo M., Dzama K. (2014). Towards a genomics approach to tick (Acari: Ixodidae) control in cattle: A review. Ticks Tick-Borne Dis..

[6] de la Fuente J. (2018). Controlling ticks and tick-borne diseases…looking forward. Ticks Tick-Borne Dis..

[7] de la Fuente J., Estrada-Peña A. (2019). Why new vaccines for the control of ectoparasite vectors have not been registered and commercialized?. Vaccines.

[8] Groot M.J., Van’t Hooft K.E. (2016). The hidden effects of dairy farming on public and environmental health in the Netherlands, India, Ethiopia, and Uganda, considering the use of antibiotics and other agro-chemicals. Front. Public Health.

[9] de la Fuente J., Almazán C., Canales M., Pérez de la Lastra J.M., Kocan K.M., Willadsen P. (2007). A ten-year review of commercial vaccine performance for control of tick infestations on cattle. Anim. Health Res. Rev..

[10] Almazán C., Tipacamu G.A., Rodriguez S., Mosqueda J., Perez de Leon A. (2018). Immunological control of ticks and tick-borne diseases that impact cattle health and production. Front. Biosci. (Landmark Ed.).

[11] Stutzer C., Richards S.A., Ferreira M., Baron S., Maritz-Olivier C. (2018). Metazoan parasite vaccines: Present status and future prospects. Front. Cell. Infect. Microbiol..

[12] Willadsen P. (2006). Vaccination against ectoparasites. Parasitology.

[13] de la Fuente J., Contreras M. (2015). Tick vaccines: Current status and future directions. Expert Rev. Vaccines.

[14] de la Fuente J., Kopáček P., Lew-Tabor A., Maritz-Olivier C. (2016). Strategies for new and improved vaccines against ticks and tick-borne diseases. Parasite Immunol..

[15] Estrada-Peña A., Szabó M., Labruna M., Mosqueda J., Merino O., Tarragona E., Venzal J.M., de la Fuente J. (2020). Towards an effective, rational and sustainable approach for the control of cattle ticks in the Neotropics. Vaccines.

[16] Schetters T., Bishop R., Crampton M., Kopáček P., Lew-Tabor A., Maritz-Olivier C., Miller R., Mosqueda J., Patarroyo J., Rodriguez-Valle M. (2016). Cattle tick vaccine researchers join forces in CATVAC. Parasites Vectors.

[17] Artigas-Jerónimo S., Villar M., Cabezas-Cruz A., Valdés J.J., Estrada-Peña A., Alberdi P., de la Fuente J. (2018). Functional evolution of Subolesin/Akirin. Front. Physiol..

[18] Adaszek L., Winiarczyk S. (2008). Molecular characterization of *Babesia canis canis* isolates from naturally infected dogs in Poland. Vet. Parasitol..

[19] Chaligiannis Ι., Fernández de Mera I.G., Papa A., Sotiraki S., de la Fuente J. (2018). Molecular identification of tick-borne pathogens in ticks collected from dogs and small ruminants from Greece. Exp. Appl. Acarol..

[20] Jones D.T., Taylor W.R., Thornton J.M. (1992). The rapid generation of mutation data matrices from protein sequences. Bioinformatics.

[21] Kumar S., Stecher G., Li M., Knyaz C., Tamura K. (2018). MEGA X: Molecular Evolutionary Genetics Analysis across computing platforms. Mol. Biol. Evol..

[22] Almazán C., Moreno-Cantú O., Moreno-Cid J.A., Galindo R.C., Canales M., Villar M., de la Fuente J. (2012). Control of tick infestations in cattle vaccinated with bacterial membranes containing surface-exposed tick protective antigens. Vaccine.

[23] Rodríguez M., Massard C.L., Henrique da Fonseca A., Fonseca Ramos N., Machado H., Labarta V., de la Fuente J. (1995). Effect of vaccination with a recombinant Bm86 antigen preparation on natural infestations of *Boophilus microplus* in grazing dairy and beef pure and cross-bred cattle in Brazil. Vaccine.

[24] Aguirre Ade A., Garcia M.V., Szabó M.P., Barros J.C., Andreotti R. (2015). Formula to evaluate efficacy of vaccines and systemic substances against three-host ticks. Int. J. Parasitol..

[25] Contreras M., Villar M., de la Fuente J. (2019). A vaccinomics approach to the identification of tick protective antigens for the control of *Ixodes ricinus* and *Dermacentor reticulatus* infestations in companion animals. Front. Physiol..

[26] Contreras M., Moreno-Cid J.A., Domingos A., Canales M., Díez-Delgado I., Pérez de la Lastra J.M., Sánchez E., Merino O., López Zavala R., Ayllón N. (2015). Bacterial membranes enhance the immunogenicity and protective capacity of the surface exposed tick Subolesin-*Anaplasma marginale* MSP1a chimeric antigen. Ticks Tick-Borne Dis..

[27] de la Fuente J., Moreno-Cid J.A., Galindo R.C., Almazán C., Kocan K.M., Merino O., Pérez de la Lastra J.M., Estrada-Peña A., Blouin E.F. (2013). Subolesin/Akirin vaccines for the control of arthropod vectors and vector-borne pathogens. Transbound. Emerg. Dis..

[28] Moreno-Cid J.A., Pérez de la Lastra J.M., Villar M., Jiménez M., Pinal R., Estrada-Peña A., Alarcón P., Delacour S., Oropeza V., Ruiz I. (2013). Control of multiple arthropod vector infestations with subolesin/akirin vaccines. Vaccine.

[29] Macqueen D.J., Johnston I.A. (2009). Evolution of the multifaceted eukaryotic akirin gene family. BMC Evol. Biol..

[30] Galindo R.C., Muñoz P.M., de Miguel M.J., Marin C.M., Blasco J.M., Gortazar C., Kocan K.M., de la Fuente J. (2009). Differential expression of inflammatory and immune response genes in rams experimentally infected with a rough virulent strain of *Brucella ovis*. Vet. Immunol. Immunopathol..

[31] Sultana H., Patel U., Sonenshine D.E., Neelakanta G. (2015). Identification and comparative analysis of subolesin/akirin ortholog from *Ornithodoros turicata* ticks. Parasites Vectors.

[32] Artigas-Jerónimo S., Pastor Comín J.J., Villar M., Contreras M., Alberdi P., León Viera I., Soto L., Cordero R., Valdés J.J., Cabezas-Cruz A. (2020). A novel combined scientific and artistic approach for advanced characterization of interactomes: The Akirin/Subolesin model. Vaccines.

[33] de la Fuente J., Moreno-Cid J.A., Canales M., Villar M., Pérez de la Lastra J.M., Kocan K.M., Galindo R.C., Almazán C., Blouin E.F. (2011). Targeting arthropod subolesin/akirin for the development of a universal vaccine for control of vector infestations and pathogen transmission. Vet. Parasitol..

[34] Contreras M., Kasaija P.D., Merino O., de la Cruz-Hernandez N.I., Gortazar C., de la Fuente J. (2019). Oral vaccination with a formulation combining *Rhipicephalus microplus* Subolesin with heat inactivated *Mycobacterium bovis* reduces tick infestations in cattle. Front. Cell. Infect. Microbiol..

[35] Merino M., Antunes S., Mosqueda J., Moreno-Cid J.A., Pérez de la Lastra J.M., Rosario-Cruz R., Rodríguez S., Domingos A., de la Fuente J. (2013). Vaccination with proteins involved in tick-pathogen interactions reduces vector infestations and pathogen infection. Vaccine.

[36] Shakya M., Kumar B., Nagar G., de la Fuente J., Ghosh S. (2014). Subolesin: A candidate vaccine antigen for the control of cattle tick infestations in Indian situation. Vaccine.

[37] Slifka M.K., Amanna I. (2014). How advances in immunology provide insight into improving vaccine efficacy. Vaccine.

[38] Yang D., Frego L., Lasaro M., Truncali K., Kroe-Barrett R., Singh S. (2016). Efficient Qualitative and Quantitative Determination of Antigen-induced Immune Responses. J. Biol. Chem..

[39] Prechl J. (2017). A generalized quantitative antibody homeostasis model: Antigen saturation, natural antibodies and a quantitative antibody network. Clin. Transl. Immunol..

[40] Canales M., Enríquez A., Ramos E., Cabrera D., Dandie H., Soto A., Falcón V., Rodríguez M., de la Fuente J. (1997). Large-scale production in *Pichia pastoris* of the recombinant vaccine Gavac against cattle tick. Vaccine.

[41] Popara M., Villar M., Mateos-Hernández L., de Mera I.G., Marina A., del Valle M., Almazán C., Domingos A., de la Fuente J. (2013). Lesser protein degradation machinery correlates with higher BM86 tick vaccine efficacy in *Rhipicephalus annulatus* when compared to *Rhipicephalus microplus*. Vaccine.

[42] Olds C.L., Mwaura S., Odongo D.O., Scoles G.A., Bishop R., Daubenberger C. (2016). Induction of humoral immune response to multiple recombinant Rhipicephalus appendiculatus antigens and their effect on tick feeding success and pathogen transmission. Parasites Vectors.

[43] Cobon G., Hungerford J., Woodrow M., Smith D., Willadsen P., de la Fuente J. (1995). Vaccination against *Boophilus microplus*: The Australian field experience. Recombinant Vaccines for the Control of Cattle Tick.

[44] de la Fuente J., Rodríguez M., Redondo M., Montero C., García-García J.C., Méndez L., Serrano E., Valdés M., Enríquez A., Canales M. (1998). Field studies and cost-effectiveness analysis of vaccination with Gavac^TM^ against the cattle tick *Boophilus microplus*. Vaccine.

[45] Contreras M., de la Fuente J. (2016). Control of *Ixodes ricinus* and *Dermacentor reticulatus* tick infestations in rabbits vaccinated with the Q38 Subolesin/Akirin chimera. Vaccine.

[46] Ghosh S., Singh N.K., Das G. (2005). Assessment of duration of immunity in crossbred cattle immunized with glycoproteins isolated from *Hyalomma anatolicum anatolicum* and *Boophilus microplus*. Parasitol. Res..

[47] Rego R.O.M., Trentelman J.J.A., Anguita J., Nijhof A.M., Sprong H., Klempa B., Hajdusek O., Tomás-Cortázar J., Azagi T., Strnad M. (2019). Counterattacking the tick bite: Towards a rational design of anti-tick vaccines targeting pathogen transmission. Parasites Vectors.

