# Supplementary material for: Vaccination with Recombinant Subolesin Antigens Provides Cross-Tick Species Protection in Bos indicus and Crossbred Cattle in Uganda
Source: Vaccines (Basel). 2020 Jun 18;8(2):319. doi: 10.3390/vaccines8020319 (PMC7350222; doi:10.3390/vaccines8020319)
Supplement: Supplementary file 1 [file vaccines-08-00319-s001.zip › Figure S1.pdf]

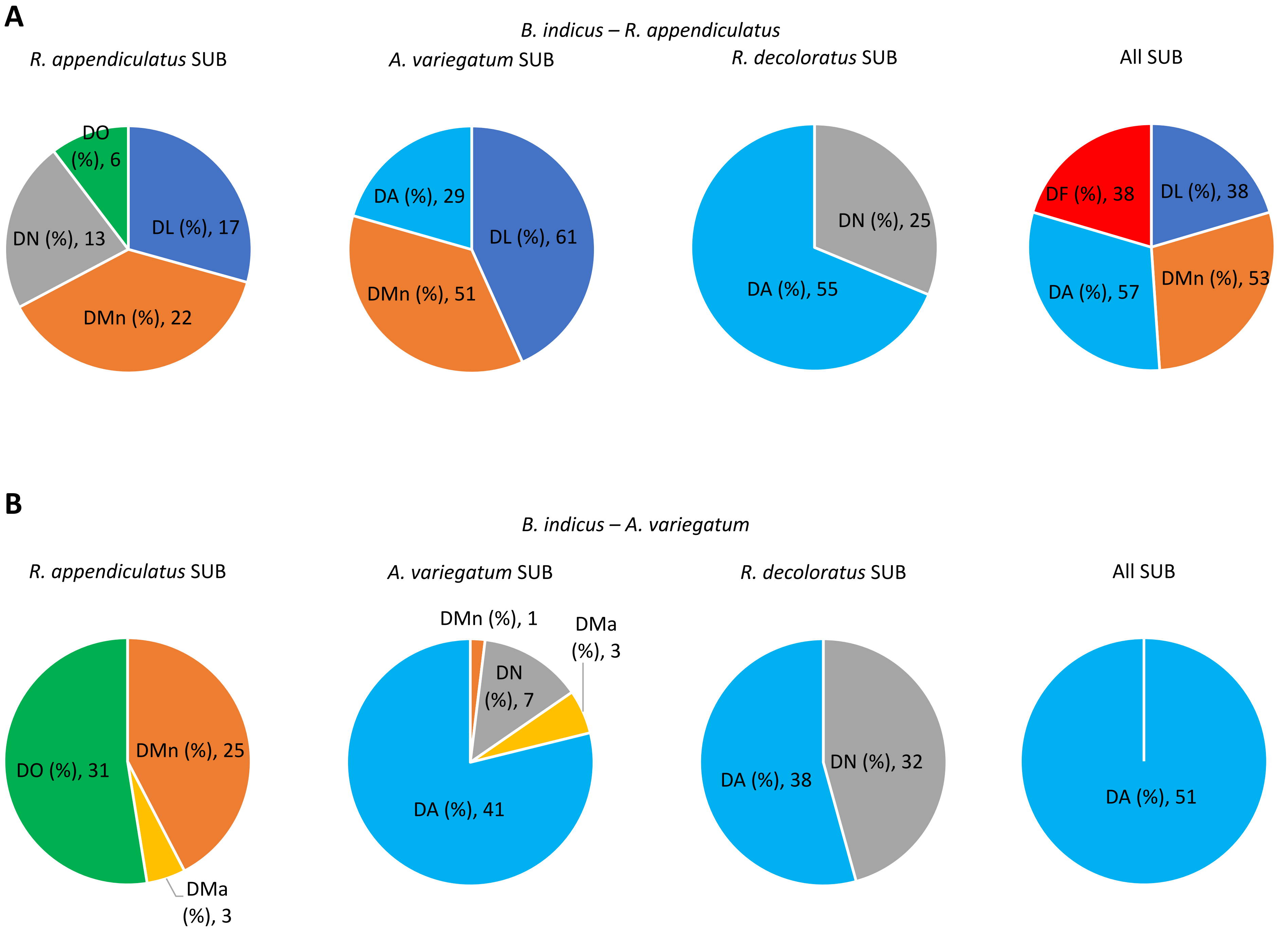
A
B. indicus – R. appendiculatus
R. appendiculatus SUB
DO (%), 6
DL (%), 17
DN (%), 13
DMn (%), 22
A. variegatum SUB
DA (%), 29
DL (%), 61
DMn (%), 51
R. decoloratus SUB
DN (%), 25
DA (%), 55
All SUB
DF (%), 38
DL (%), 38
DMn (%), 53
DA (%), 57
B
B. indicus – A. variegatum
R. appendiculatus SUB
DMn (%), 25
DO (%), 31
DMa (%), 3
A. variegatum SUB
DMn (%), 1
DMa (%), 3
DN (%), 7
DA (%), 41
R. decoloratus SUB
DN (%), 32
DA (%), 38
All SUB
DA (%), 51

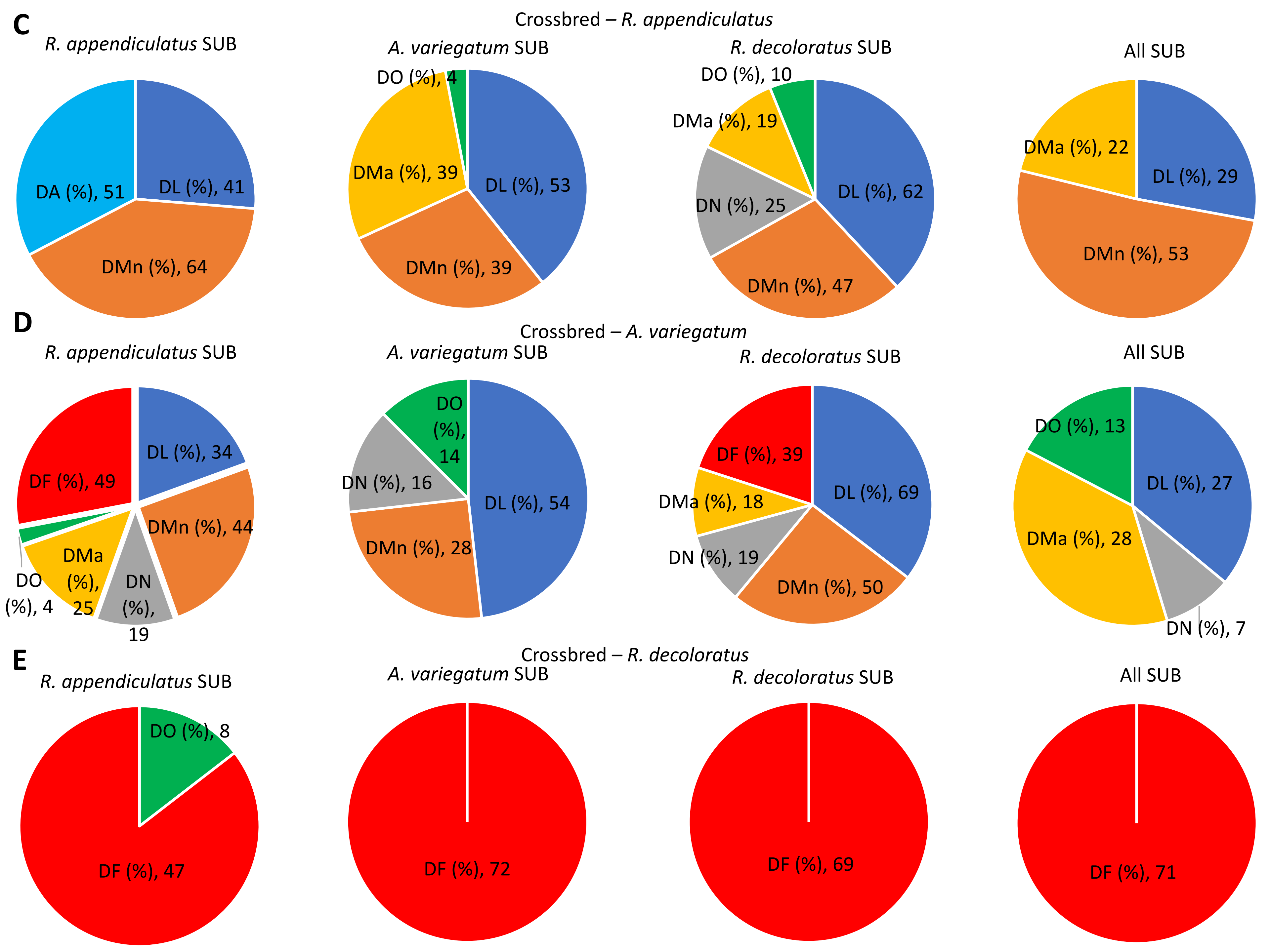

C
Crossbred – *R. appendiculatus*
R. appendiculatus SUB
DA (%), 51
DL (%), 41
DMn (%), 64
A. variegatum SUB
DO (%), 4
DMa (%), 39
DL (%), 53
DMn (%), 39
R. decoloratus SUB
DO (%), 10
DMa (%), 19
DN (%), 25
DL (%), 62
DMn (%), 47
All SUB
DMa (%), 22
DL (%), 29
DMn (%), 53
D
Crossbred – *A. variegatum*
R. appendiculatus SUB
DL (%), 34
DF (%), 49
DMn (%), 44
DMa (%), 25
DO (%), 4
DN (%), 19
A. variegatum SUB
DO (%), 14
DN (%), 16
DL (%), 54
DMn (%), 28
R. decoloratus SUB
DF (%), 39
DL (%), 69
DMa (%), 18
DN (%), 19
DMn (%), 50
All SUB
DO (%), 13
DL (%), 27
DMa (%), 28
DN (%), 7
E
Crossbred – *R. decoloratus*
R. appendiculatus SUB
DO (%), 8
DF (%), 47
A. variegatum SUB
DF (%), 72
R. decoloratus SUB
DF (%), 69
All SUB
DF (%), 71

Figure S1. Effect of vaccination with SUB on different tick developmental stages. (A) *B. indicus – R. appendiculatus.* (B) *B. indicus – A. variegatum*. (C) Crossbred – *R. appendiculatus*. (D) Crossbred-*A. variegatum*. (E) Crossbred – *R. decoloratus*. The effect of vaccination on different tick developmental stages was used to evaluate vaccine E. The tick developmental stages included the number of engorged larvae (DL), nymphs (DN) and adult female ticks (DA), molting of tick larvae (DMn) and nymphs (DMa), oviposition (DO) and fertility (DF). The percent reduction for each developmental stage is shown on the graphs. Only parameters with statistically significant differences (Chi-square test; $p < 0.05$, $n = 4$ biological replicates; Data S1) are shown and were included in the vaccine E calculation.
